# S100A8/A9 promotes endometrial fibrosis via regulating RAGE/JAK2/STAT3 signaling pathway

**DOI:** 10.1038/s42003-024-05814-5

**Published:** 2024-01-22

**Authors:** Xing Xin, Hao Liu, Siwen Zhang, Pingping Li, Xinyang Zhao, Xudong Zhang, Shuyu Li, Shanshan Wu, Fujie Zhao, Jichun Tan

**Affiliations:** 1 Center of Reproductive Medicine, Department of Obstetrics and Gynecology, Shengjing Hospital of China Medical University, No. 39 Huaxiang Road, Tiexi District, 110022 Shenyang, China; 2 Key Laboratory of Reproductive Dysfunction Disease and Fertility Remodeling of Liaoning Province, No. 39 Huaxiang Road, Tiexi District, 110022 Shenyang, China; 3 State Key Laboratory of Robotics, Shenyang Institute of Automation, Chinese Academy of Sciences, Shenyang, China; 4 Institutes for Robotics and Intelligent Manufacturing, Chinese Academy of Sciences, Shenyang, China; 5 Key Laboratory of Minimally Invasive Surgical Robot, Liaoning Province, Shenyang, China; 6 Obstetrics and Gynecology Department, Shengjing Hospital of China Medical University, No. 36 Sanhao Street, Heping District, 110022 Shenyang, China

**Keywords:** Infertility, Mesenchymal stem cells

## Abstract

Intrauterine adhesion (IUA) is characterized by endometrial fibrosis. S100A8/A9 plays an important role in inflammation and fibroblast activation. However, the role of S100A8/A9 in IUA remains unclear. In this study, we collect normal and IUA endometrium to verify the expression of S100A8/A9. Human endometrial stromal cells (hEnSCs) are isolated to evaluate fibrosis progression after S100A8/A9 treatment. A porcine IUA model is established by electrocautery injury to confirm the therapeutic effect of menstrual blood-derived stromal cells (MenSCs) on IUA. Our study reveals increased S100A8/A9 expression in IUA endometrium. S100A8/A9 significantly enhances hEnSCs proliferation and upregulates fibrosis-related and inflammation-associated markers. Furthermore, S100A8/A9 induces hEnSCs fibrosis through the RAGE-JAK2-STAT3 pathway. Transplantation of MenSCs in a porcine IUA model notably enhances angiogenesis, mitigates endometrial fibrosis and downregulates S100A8/A9 expression. In summary, S100A8/A9 induces hEnSCs fibrosis via the RAGE-JAK2-STAT3 pathway, and MenSCs exhibit marked effects on endometrial restoration in the porcine IUA model.

## Abstract (web summary)

A study on clinical tissue specimens and a porcine model reveals that S100A8/A9 mediates endometrial stromal cell fibrosis and IUA by activating the RAGE-JAK2-STAT3 pathway. These effects can be ameliorated through MenSCs transplantation.

## Introduction

IUA is a common cause of female infertility, characterized by endometrial fibrosis resulting from damage to the endometrial basal layer caused by trauma or infection^1^. This damage leads to the massive proliferation and differentiation of hEnSCs into myofibroblasts, resulting in the secretion and deposition of ECM, possibly leading to partial or complete occlusion of the uterine cavity^2^. However, the molecular mechanism underlying IUA formation is still not well-understood.

S100A8/A9, a member of the S100 calcium-binding protein family, is mainly expressed in myeloid cells such as neutrophils and is actively released during inflammation^3^. S100A8/A9 stimulates leukocyte recruitment and cytokine secretion, and under stress conditions, it activates inflammatory pathways and leads to the secretion of multiple cytokines that sustain or exacerbate inflammation^4^. Moreover, S100A8/A9 promotes the proliferation and differentiation of fibroblasts into myofibroblasts, leading to the secretion and deposition of ECM^5^. However, the role and mechanism of S100A8/A9 in IUA development have not yet been investigated.

In stress conditions, the binding of S100A8/A9 to RAGE activates inflammatory pathways, subsequently triggering the release of various pro-inflammatory factors and cytokines that perpetuate the inflammatory response^4^. RAGE is a pattern-recognition receptor capable of interacting with diverse endogenous ligands, thereby initiating multiple signaling cascades, including the JAK/STAT pathway, which has been implicated in the pathogenesis of inflammatory responses^6^. Based on this knowledge, we postulated that S100A8/A9, by binding to RAGE, activates the JAK2/STAT3 signaling pathway. This activation, in turn, leads to the release of pro-inflammatory factors and cytokines, facilitates the proliferation and activation of fibroblasts, enhances the expression and accumulation of ECM, and ultimately culminates in the development of fibrosis.

The present standard surgical procedure for clinical treatment of IUA is the transcervical resection of adhesion, which aims to restore the anatomical structure of the uterine cavity^7^. However, this procedure often results in fibrous scars that cause re-adhesion. Recent studies have demonstrated the effectiveness of MenSCs transplantation in treating IUA. These stem cells possess multilineage potential and have been extensively studied in regenerative medicine^8^. In previous clinical and animal studies, autologous MenSCs transplantation was shown to significantly accelerate endometrial repair and promote fertility recovery in IUA patients^9^ and rats^10^, respectively.

Our investigation reveals a significant elevation of S100A8/A9 in IUA endometrium, predominantly derived from neutrophils. In vitro, S100A8/A9 promotes hEnSCs proliferation, inflammatory response, and induces the differentiation of hEnSCs into myofibroblasts via the RAGE-JAK2-STAT3 signaling pathway. In a porcine IUA model, electrocautery injury increases S100A8/A9, RAGE, p-JAK2, and p-STAT3 expression. MenSCs treatment reduces these markers, suggesting a potential role in IUA prevention and treatment by modulating inflammation, suppressing S100A8/A9 secretion, and inhibiting ECM deposition.

## Results

### S100A8/A9 was upregulated in the endometrium of IUA patients

**Fig. 1:**
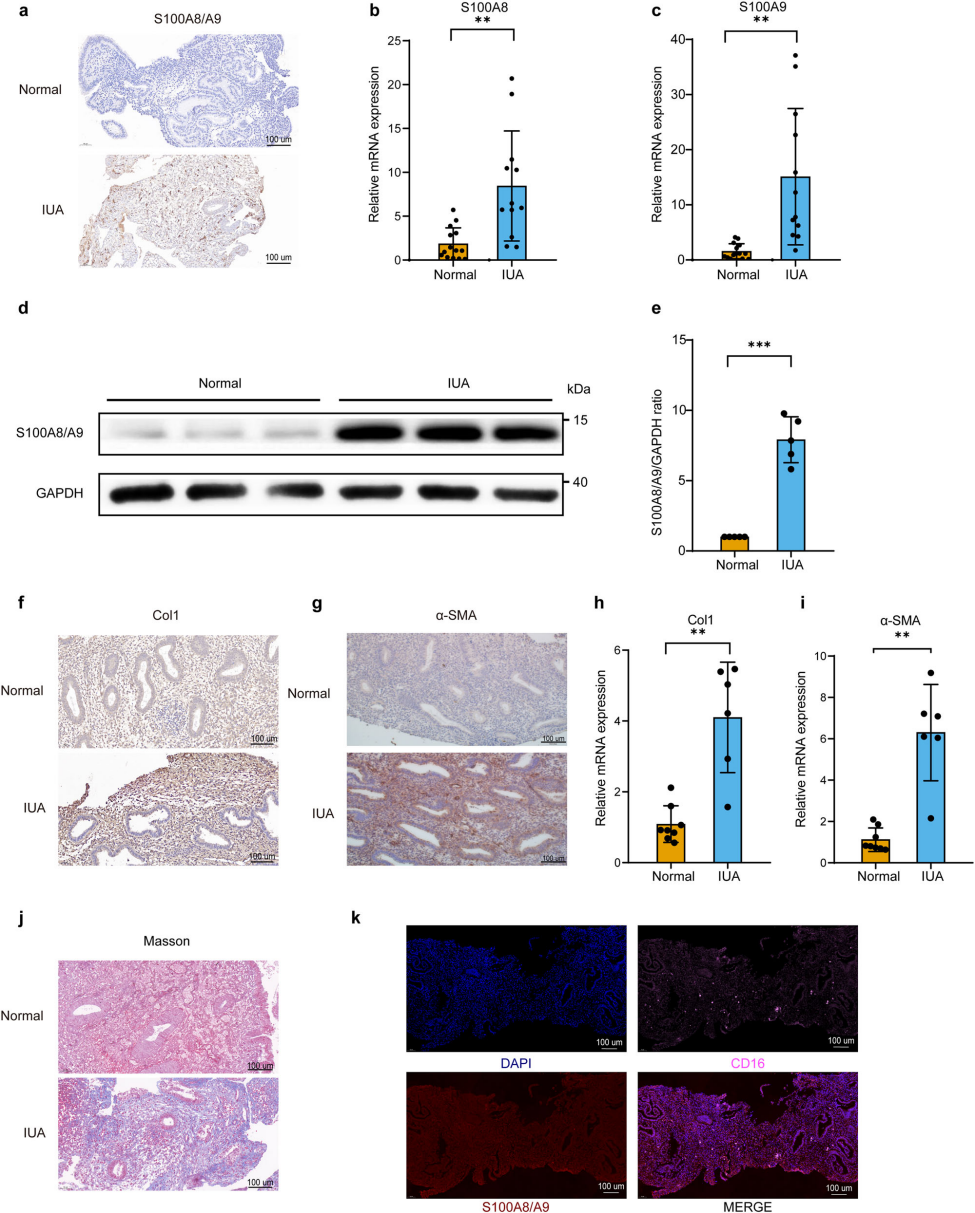
S100A8/A9, Col1, and α-SMA is upregulated in the endometrium of patients with IUA. **a** Representative immunohistochemistry images of S100A8/A9 in endometrial tissue sections from normal subjects and patients with IUA are shown. Scale bar = 100 μm. **b**, **c** The expression levels of *S100A8* and *S100A9* mRNA in endometrial tissues were evaluated using RT-PCR, *n* = 14 for the normal group, *n* = 12 for the IUA group, unpaired *t* test with Welch’s correction. **d**, **e** The protein expression of S100A8/A9 in endometrial tissue from normal subjects and patients with IUA was determined by western blot analysis, *n* = 5 per group, unpaired *t* test with Welch’s correction. **f**, **g** Immunohistochemistry staining of Col1 and α-SMA in endometrial tissue sections from normal subjects and patients with IUA. Scale bar = 100 μm. **h**, **i** The mRNA levels of *Col1* and *α-SMA* in endometrial tissues were measured by RT-PCR, *n* = 8 for the normal group, *n* = 6 for the IUA group, unpaired *t* test with Welch’s correction. **j** Masson staining of endometrial tissue sections from normal subjects and patients with IUA. Scale bar = 100 μm. **k** Immunofluorescence staining of CD16, S100A8/A9 in endometrial tissue sections from IUA patients (blue: DAPI, pink: CD16, red: S100A8/A9, Scale bar = 100 μm). Data are presented as means ± SD. *P < 0.05, **P < 0.01, ***P < 0.001 compared with the indicated groups.

Real-time PCR, immunohistochemical staining and Western blot results demonstrated significantly elevated mRNA and protein expression levels of S100A8/A9, as well as the fibrosis-related markers Type I collagen (Col1) and alpha-smooth muscle actin (α-SMA) in the endometrium of the IUA group compared to those in the normal group (Fig. 1a–i). Masson staining revealed numerous dark blue and disorganized fibrous structures in the endometrium of IUA patients (Fig. 1j). To determine the primary source of S100A8/A9 in the endometrium of IUA patients, immunofluorescence staining was employed to detect the co-localization of S100A8/A9 and the neutrophil marker CD16. The results indicated a high degree of co-localization between S100A8/A9-positive cells and CD16-positive cells in the endometrial tissues of IUA patients (Fig. 1k), suggesting that S100A8/A9 may be primarily secreted by neutrophils.

### S100A8/A9 promoted the proliferation and fibrosis of hEnSCs in vitro

Subsequently, we investigated the impact of S100A8/A9 on hEnSCs in vitro. The hEnSCs used in this study were isolated and cultured according to our previously published protocol using endometrial tissue obtained from volunteers^11^. Primary cultured hEnSCs display rapid adhesion, featuring large spindle-shaped cell bodies, centered oval nuclei (Supplementary Fig. 1a), and Vimentin-positive (Supplementary Fig. 1b), α-SMA-negative (Supplementary Fig. 1c) immunofluorescence, confirming their identity.

**Fig. 2:**
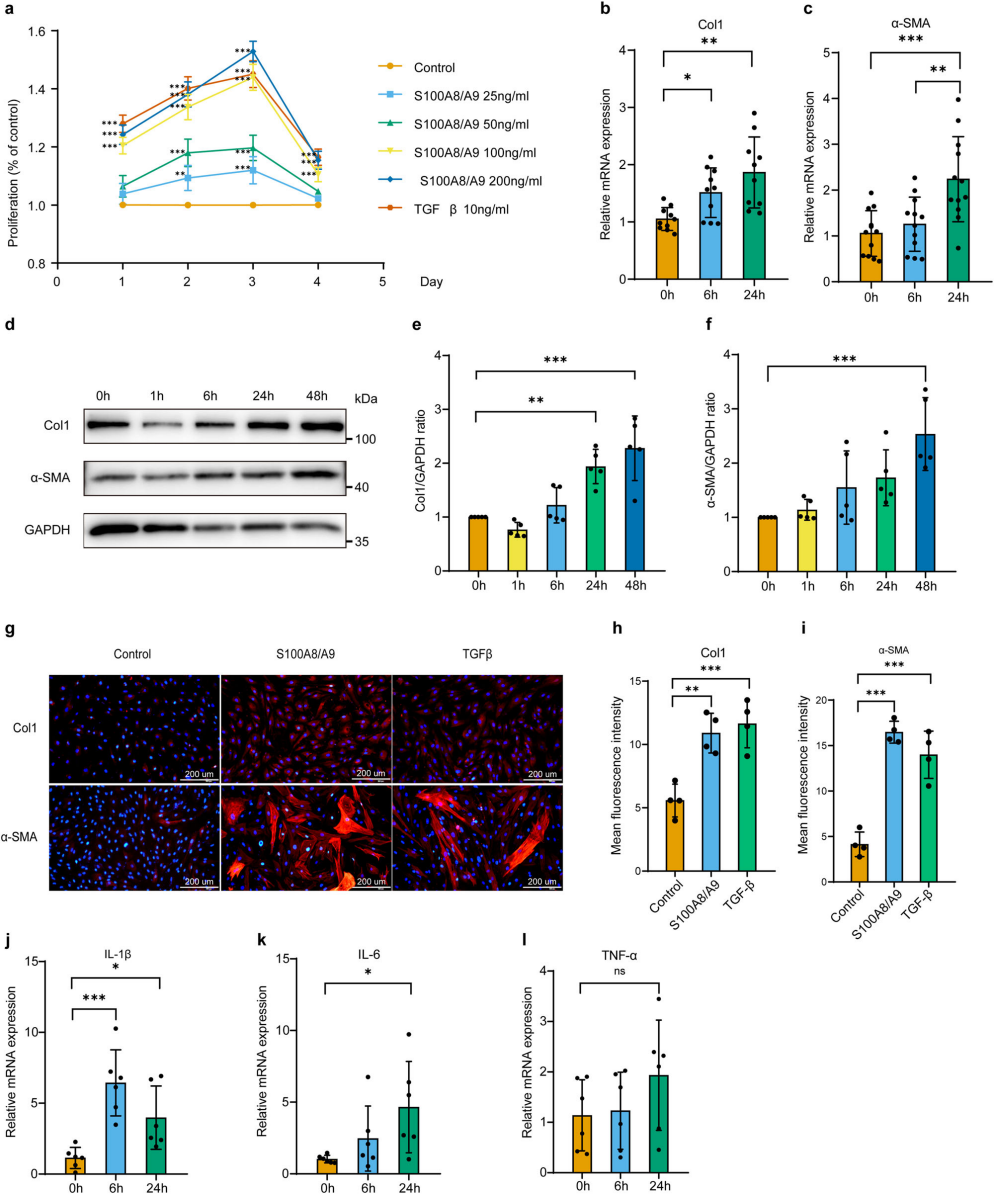
S100A8/A9 promotes the proliferation and fibrosis of hEnSCs in vitro. **a** hEnSCs were exposed to various concentrations (25/50/100/200 ng/mL) of S100A8/A9 or 10 ng/mL TGF-β1 for 1–4 days, and cell proliferation was assessed using the CCK-8 assay, *n* = 4 per group, two-way ANOVA with Tukey’s multiple comparison test. **b** Following treatment with 100 ng/mL S100A8/A9 for 6 or 24 h, the expression levels of *Col1* mRNA in hEnSCs were measured by RT-PCR, *n* = 10 per group, Welch’s ANOVA test with Tamhane’s T2 multiple comparisons test. **c** Following treatment with 100 ng/mL S100A8/A9 for 6 or 24 h, the expression levels of *α-SMA* mRNA in hEnSCs were measured by RT-PCR, *n* = 12 per group, ordinary one-way ANOVA with Tukey’s multiple comparison test. **d**–**f** After treatment with 100 ng/mL S100A8/A9 for 1, 6, 24, and 48 h, the protein expression of Col1 and α-SMA in hEnSCs was determined by western blot analysis, *n* = 5 per group, ordinary one-way ANOVA with Dunnett’s multiple comparison test. **g**–**i** Following treatment with 100 ng/mL S100A8/A9 or 10 ng/mL TGF-β1 for 48 h, the protein expression of Col1 and α-SMA in hEnSCs was detected by immunofluorescence staining (blue: DAPI, red: Col1 or α-SMA, Scale bar = 200 μm), *n* = 4 per group, ordinary one-way ANOVA with Dunnett’s multiple comparison test. **j**–**l** After treatment with 100 ng/mL S100A8/A9 for 6 and 24 h, the expression levels of *IL-1β*, *IL-6*, and *TNF-α* mRNA in hEnSCs were measured by RT-PCR, *n* = 6 per group, ordinary one-way ANOVA with Dunnett’s multiple comparison test. Data are presented as means ± SD. *P < 0.05, **P < 0.01, ***P < 0.001 compared with indicated groups. ns, not significant (*P* > 0.05).

In this study, the effects of S100A8/A9 on the proliferation and fibrosis of hEnSCs were investigated. To evaluate the impact of S100A8/A9 on hEnSCs proliferation, cells were exposed to varying concentrations of the protein, and proliferation was measured using the CCK-8 assay. In our prior investigation, the plasma concentration of S100A8/A9 in 39 IUA patients was determined by ELISA to be 97.3 ± 46.7 ng/ml (with a range of 48.9–311.3 ng/ml). Consequently, concentration gradients of 25 ng/ml, 50 ng/ml, 100 ng/ml, and 200 ng/ml were chosen for CCK-8 assay. It was shown that treatment with 100 ng/mL of S100A8/A9 significantly increased the proliferation of hEnSCs in vitro, with the most significant effect observed on the third day (Fig. 2a). Interestingly, the proliferative effect of S100A8/A9 at 100 ng/mL was comparable to that of the positive control, TGF-β (10 ng/ml). Therefore, subsequent experiments aimed at stimulating hEnSCs were carried out using 100 ng/mL of S100A8/A9.

To investigate the potential role of S100A8/A9 in promoting fibrosis, hEnSCs were treated with 100 ng/mL of S100A8/A9 (Novoprotein, China), and the expressions of Col1 and α-SMA were analyzed by RT-PCR, western blot and IF staining. Results revealed that the relative mRNA (Fig. 2b, c) and protein (Fig. 2d–i) expression levels of Col1 and α-SMA were significantly increased after S100A8/A9 stimulation.

Furthermore, to assess the role of S100A8/A9 in promoting pro-inflammatory cytokine secretion by hEnSCs, RT-PCR was performed to analyze the gene expression of *IL-1β*, *interleukin-6 (IL-6)*, and *TNFα*. The results showed a significant increase in the expression of *IL-1β* and *IL-6* after 6 or 24 h of S100A8/A9 incubation (Fig. 2j, k). Although *TNF-α* mRNA expression exhibited an upward trend, the results were not statistically significant (Fig. 2l).

These findings suggest that S100A8/A9 may play a role in promoting inflammation and fibrosis in the endometrium.

### S100A8/A9mediated hEnSCs fibrosis through RAGE/JAK2/STAT3 pathway

**Fig. 3:**
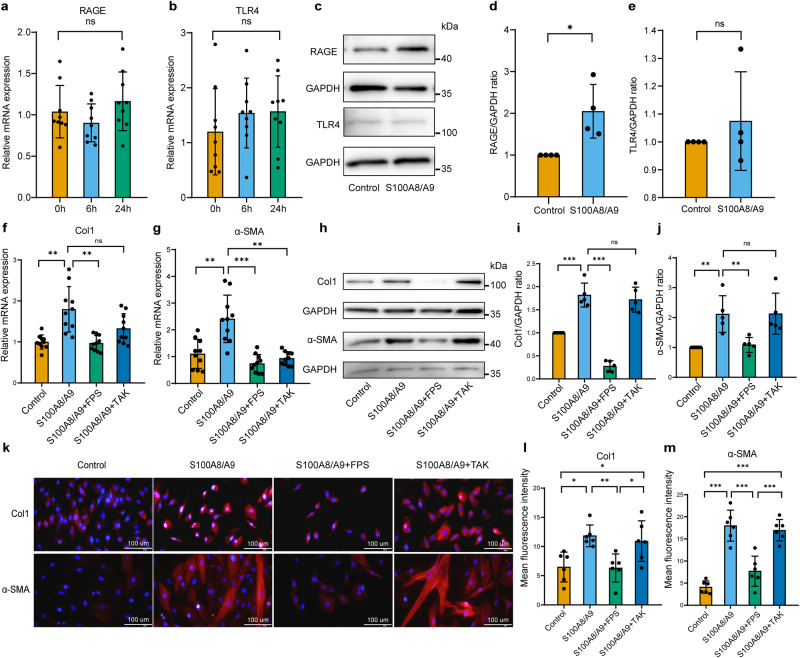
S100A8/A9 requires RAGE to induce differentiation of hEnSCs into myofibroblasts and promote fibrosis formation. After treatment of hEnSCs with S100A8/A9 for 6 and 24 h, the expression levels of *RAGE* and *TLR4* mRNA were measured by RT-PCR, *n* = 9 per group, ordinary one-way ANOVA with Dunnett’s multiple comparison test (**a**, **b**), while the protein expression levels of RAGE and TLR4 were determined by western blot analysis, *n* = 4 per group, unpaired *t* test with Welch’s correction (**c**–**e**). hEnSCs were pretreated with 10 μg/mL FPS-ZM1 or 10 μg/mL TAK-242 for 1 h, followed by S100A8/A9 stimulation for 48 h. The expression levels of *Col1* and *α-SMA* mRNA were measured by RT-PCR, *n* = 10 per group, Welch’s ANOVA test with Tamhane’s T2 multiple comparisons test (**f**, **g**), while the protein expression levels of Col1 and α-SMA were detected by western blot analysis, *n* = 5 per group, ordinary one-way ANOVA with Dunnett’s multiple comparison test (**h**–**j**) and immunofluorescence staining (red: Col1 or α-SMA, blue: DAPI, scale bar = 100 μm), *n* = 6 per group, ordinary one-way ANOVA with Tukey’s multiple comparison test (**k**–**m**). Data are presented as means ± SD. *P < 0.05, **P < 0.01, ***P < 0.001 compared with indicated groups. ns, not significant (*P* > 0.05).

First, we examined whether S100A8/A9 mediates fibrosis by affecting TLR4 or RAGE in hEnSCs. The results indicate that the mRNA expression of *RAGE* and *TLR4* in hEnSCs was barely affected by S100A8/A9 (Fig. 3a, b), while the protein expression of RAGE was significantly increased, and the protein expression of TLR4 was not affected (Fig. 3c–e). Further investigation into the role of RAGE in S100A8/A9-induced fibrosis formation and differentiation of hEnSCs into myofibroblasts involved pretreating hEnSCs with 10 μg/ml FPS-ZM1(RAGE blocker) or 10 μg/ml TAK-242(TLR4 blocker) for 1 h, followed by S100A8/A9 stimulation for 48 h. The expression levels of Col1 and α-SMA mRNA and protein were evaluated using RT-PCR, western blot, and immunofluorescence techniques. The results indicate that FPS-ZM1 significantly inhibited the promotion of S100A8/A9 on the expression of Col1 and α-SMA in hEnSCs, while TAK-242 had no inhibitory effect (Fig. 3f–m), suggesting that S100A8/A9 requires RAGE to induce differentiation of hEnSCs into myofibroblasts and promote fibrosis formation.

**Fig. 4:**
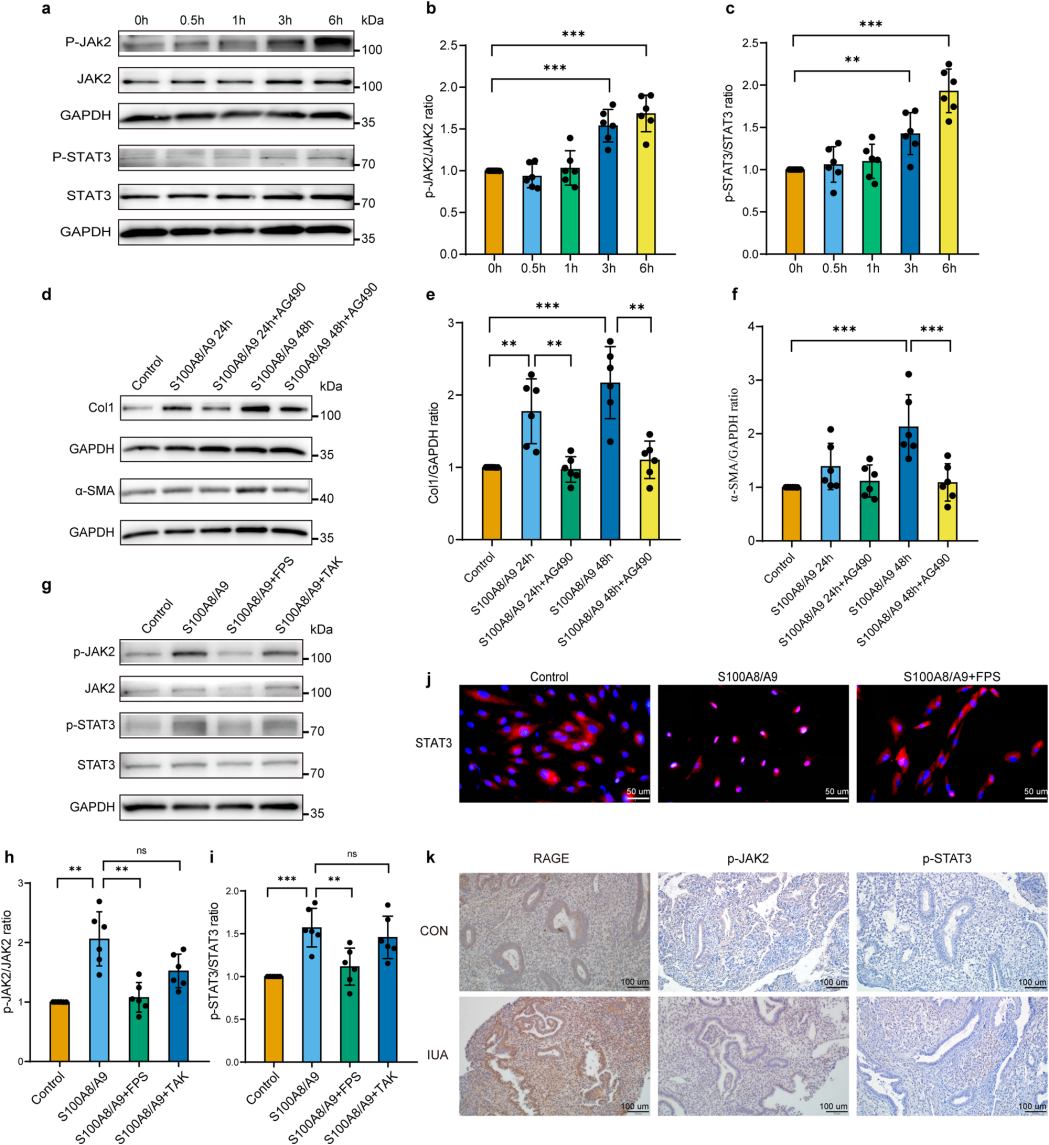
S100A8/A9 activates the JAk2/STAT3 signaling pathway through RAGE. **a**–**c** The expression of JAK2, p-JAK2, STAT3, and p-STAT3 was detected by western blot after treating hEnSCs with S100A8/A9 for 0.5 h, 1 h, 3 h, and 6 h, *n* = 6 per group, ordinary one-way ANOVA with Dunnett’s multiple comparison test. **d**–**f** Pretreating hEnSCs with AG490 for 1 h followed by S100A8/A9 treatment for 24 h and 48 h, the expression of Col1 and α-SMA was detected by western blot, *n* = 6 per group, ordinary one-way ANOVA with Tukey’s multiple comparison test. **g**–**i** Pretreating hEnSCs with FPS-ZM1 or TAK-242 for 1 h followed by S100A8/A9 stimulation for 6 h, the expression of JAK2, p-JAK2, STAT3, and p-STAT3 was detected by western blot, *n* = 6 per group, ordinary one-way ANOVA with Dunnett’s multiple comparison test. **j** Immunofluorescence was used to detect the expression of STAT3 in hEnSCs. red: STAT3, blue: DAPI, scale bar = 50 μm. **k** Representative immunohistochemistry images of RAGE, p-JAK2, p-STAT3 in endometrial tissue sections from normal subjects and patients with IUA are shown, scale bar = 100 μm. Data are presented as means ± SD. *P < 0.05, **P < 0.01, ***P < 0.001 compared with indicated groups. ns, not significant (*P* > 0.05).

Second, we explored the potential role of the JAK2/STAT3 signaling pathway in fibrosis, specifically in response to S100A8/A9 treatment in hEnSCs. Western blotting was performed to investigate the protein expression of JAK2, p-JAK2, STAT3, and p-STAT3 in hEnSCs treated with S100A8/A9 (100 ng/ml). The results indicated that phosphorylation of JAK2 (Tyr1007) and STAT3 (Tyr705) significantly increased after S100A8/A9 treatment for 3 h (Fig. 4a–c). To further investigate the role of JAK2/STAT3 signaling in S100A8/A9-induced fibrosis and differentiation of hEnSCs into myofibroblasts, hEnSCs were pretreated with AG490 (a JAK2/STAT3 pathway inhibitor) for 1 h, followed by S100A8/A9 treatment for 24 and 48 h. The results indicated that AG490 significantly inhibited the promoting effect of S100A8/A9 on the expression of Col1 and α-SMA in hEnSCs (Fig. 4d–f), suggesting that the JAK2/STAT3 pathway is required for S100A8/A9-induced fibrosis formation and differentiation of hEnSCs into myofibroblasts.

Third, we investigated whether RAGE and TLR4 are involved in the activation process of the JAK2/STAT3 signaling pathway. hEnSCs were pretreated with FPS-ZM1 or TAK-242 for 1 h, followed by S100A8/A9 stimulation. Western blotting analysis was performed to assess the phosphorylation levels of JAK2 and STAT3. The results indicated that FPS-ZM1 significantly inhibited the promotion by S100A8/A9 on the phosphorylation of JAK2 and STAT3 in hEnSCs, while TAK-242 had no such inhibitory effect (Fig. 4g–i), suggesting that S100A8/A9 requires RAGE to induce the activation of the JAK2/STAT3 signaling pathway.

In addition, following S100A8/A9 stimulation, a significant aggregation of STAT3 in the nucleus was observed with enhanced red fluorescence in comparison to the control group. However, treatment with FPS-ZM1 inhibited the nuclear translocation of STAT3 (Fig. 4j). This indicated that S100A8/A9 requires RAGE for promoting the nuclear translocation of STAT3. The collective findings suggest that S100A8/A9 contributes to fibrosis in hEnSCs by activating the RAGE/JAK2/STAT3 pathway.

Finally, we employed immunohistochemistry to evaluate the expression of RAGE, p-JAK2, and p-STAT3 in paraffin sections of endometrial tissues from both normal subjects and IUA patients (Fig. 4k). Our findings revealed an elevated expression of RAGE, p-JAK2, and p-STAT3 in the endometrial tissues of IUA patients compared with normal subjects.

### The electrocautery injury successfully constructed a porcine model of IUA

**Fig. 5:**
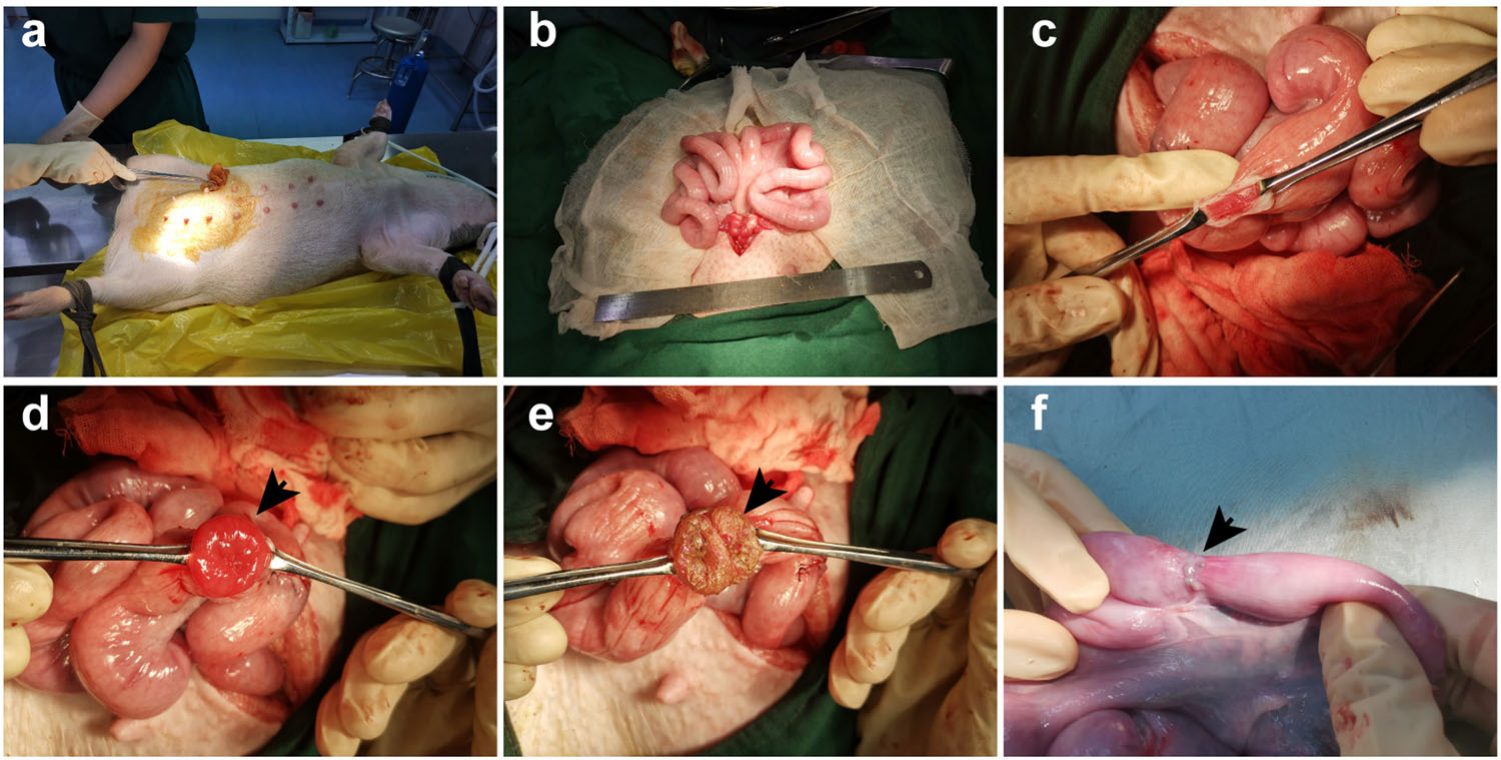
Electrocautery injury method constructs a porcine model of IUA. **a** The Bama miniature pig was placed in a supine position on the operating table after general anesthesia. **b** A midline abdominal incision was made to expose the uterus. **c** The uterine muscle layer was incised on the opposite side of the uterine mesangium. **d** The endometrium was incised to expose the uterine cavity. **e** The endometrium was electrocauterized. **f** Adhesion conditions were observed 35 days postoperatively.

We have successfully established an IUA model in Bama miniature pigs utilizing the electrocautery injury technique. The Bama miniature pig was placed in a supine position on the operating table after general anesthesia (Fig. 5a). The uterine cornua, characterized by their pliable and stretchy nature, extend bilaterally in a Y-shaped configuration, measuring approximately 40–50 cm in length and 1.5–2 cm in diameter (Fig. 5b). Upon electrocautery injury, the previously soft and pinkish endometrial tissue (Fig. 5c, d) transforms into a yellow-brown appearance, accompanied by tissue edema and hemorrhage (Fig. 5e). Following 35 days post-injury, a significant reduction in the uterine cavity diameter at the injury site was observed (Fig. 5f).

**Fig. 6:**
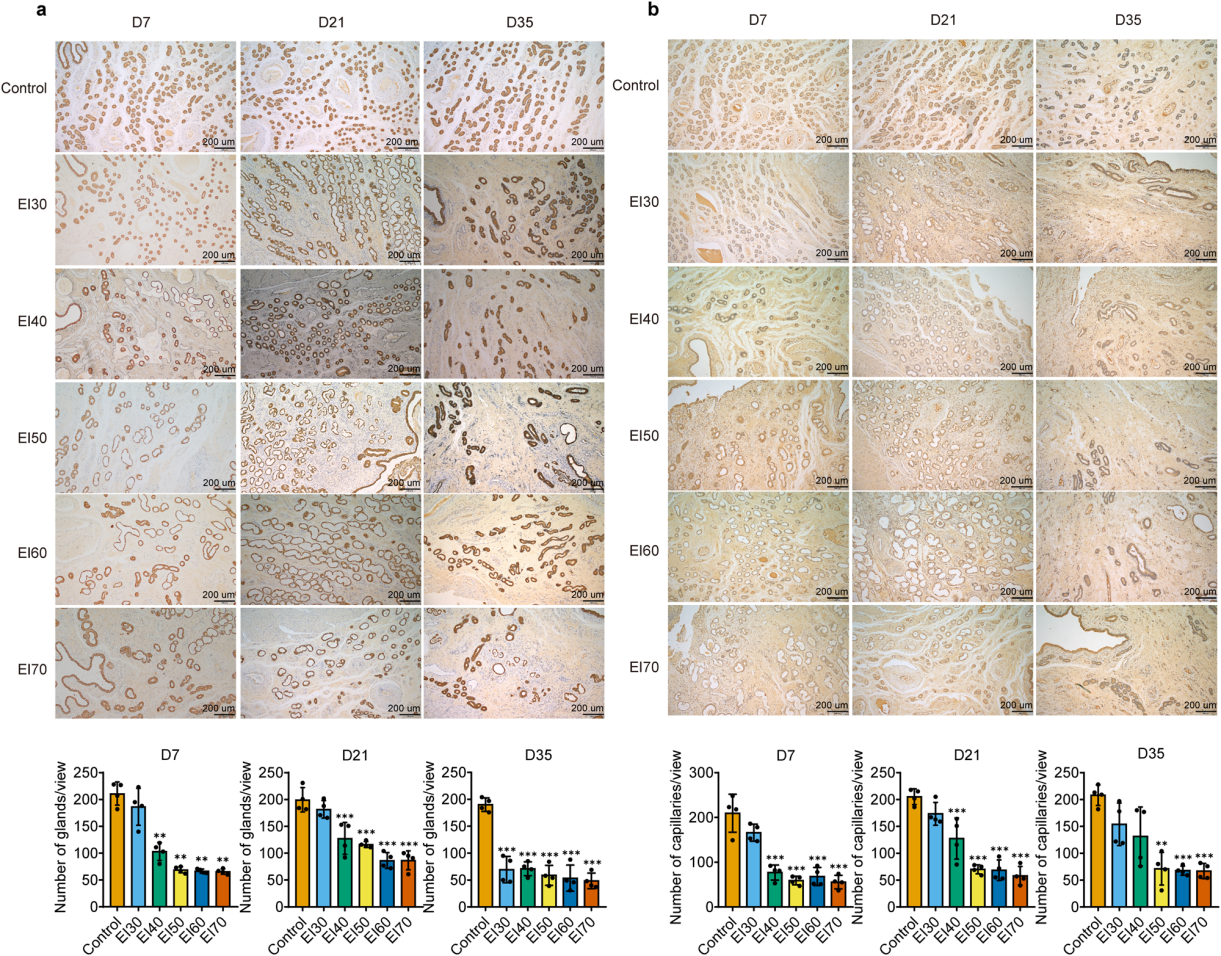
Electrocautery injury reduces the number of glands and capillaries in porcine endometrial tissue. Different electrical intensities were used to injure the endometrium, and tissue samples were taken at 7, 21, and 35 days post surgery. Paraffin sections were prepared and stained with CK18 to detect the number of endometrial glands (**a**) and vWF to detect the number of endometrial capillaries (**b**). Scale bar = 200 μm, *n* = 4 per group, ordinary one-way ANOVA with Dunnett’s multiple comparison test or Welch’s ANOVA test with Dunnett’s T3 multiple comparisons test. Data are presented as means ± SD. *P < 0.05, **P < 0.01, ***P < 0.001 compared with indicated groups.

**Fig. 7:**
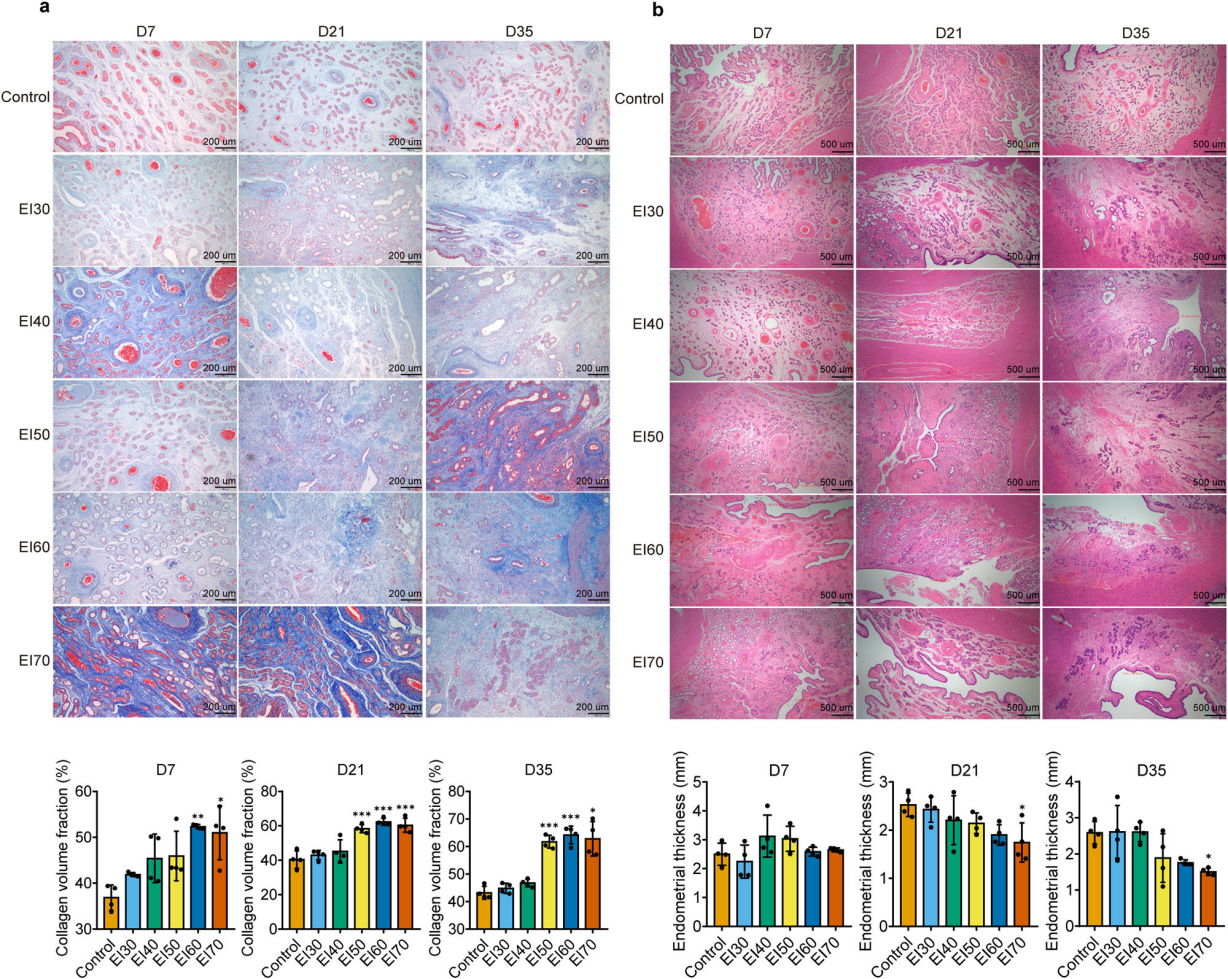
Electrocoagulation injury increases the amount of collagen in the endometrium and reduces its thickness. Different electrical intensities were used to injure the endometrium, and tissue samples were taken at 7, 21, and 35 days post surgery. Paraffin sections were prepared and stained with Masson to detect the Collagen volume fraction (scale bar =  200 μm) (**a**) and HE to detect the thickness of the endometrium (scale bar = 500 μm) (**b**), *n* = 4 per group, ordinary one-way ANOVA with Dunnett’s multiple comparison test or Welch’s ANOVA test with Dunnett’s T3 multiple comparisons test. Data are presented as means ± SD. *P < 0.05, **P < 0.01, ***P < 0.001 compared with indicated groups.

To investigate the effects of electrocautery injury on the endometrium, we cauterized the endometrium with varying intensities ranging from 30 W to 70 W. Paraffin sections of the endometrium were obtained at 7, 21, and 35 days after electrocauterization and subjected to HE staining, Masson staining, CK18 and vWF immunohistochemical staining. CK18 and vWF staining were utilized to evaluate endometrial glands and capillaries in different groups. Our results indicate that endometrial glands in the normal control group were regular in shape and abundant in number, while the number of endometrial glands significantly decreased at 7, 21, and 35 days post surgery when the electrocautery intensity reached 40 W and above (Fig. 6a). Similarly, the number of endometrial capillaries was significantly lower in the electrocautery groups with an intensity of 50 W and above compared to the control group (Fig. 6b). Masson staining results demonstrated an upward trend in the area of dark blue in each group compared to the control group, with a significant increase observed in the groups of 50 W and above (Fig. 7a). HE staining revealed that the uterine cavity had a uniform thickness and regular shape of the endometrium. Seven days after electrocauterization, the endometrial thickness of the EI40-EI70 groups increased in comparison to the control group, possibly linked to acute endometrial edema following electrocautery injury. In contrast, after 21 and 35 days of electrocauterization, the endometrial thickness of each electrocautery group was reduced relative to the control group, and the EI70 group displayed a significant reduction in endometrial thickness (Fig. 7b). These findings suggest that the endometrium of pigs can be effectively damaged when the electrocautery intensity reaches 70 W, which was used in subsequent experiments.

### MenSCs treatment promoted the repair of endometrial injury in a porcine model of IUA

**Fig. 8:**
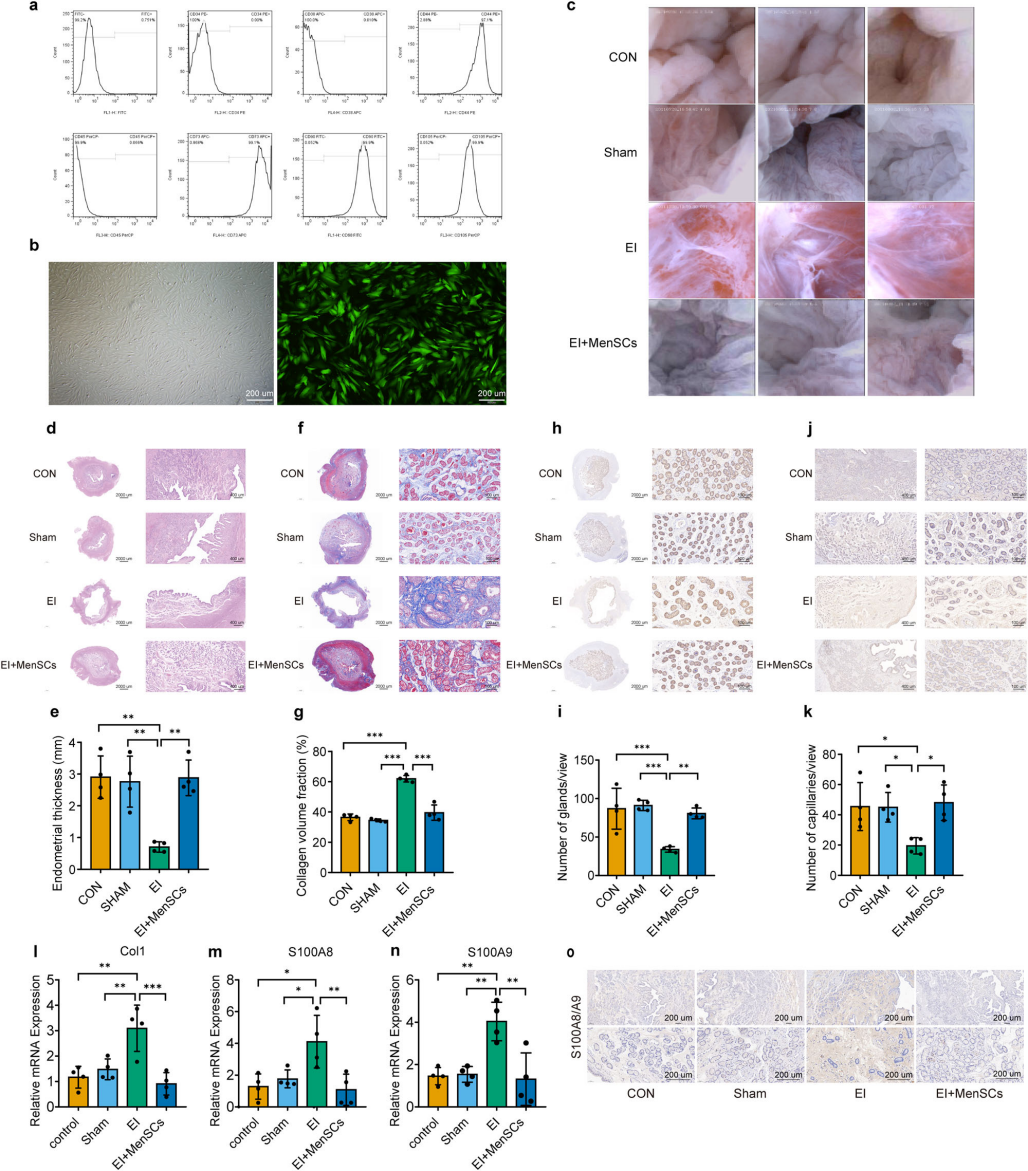
MenSCs treatment promotes the repair of endometrial injury in a porcine model of IUA. **a** Flow cytometry analysis of surface markers of MenSCs. **b** Morphology of MenSCs under light microscope and fluorescence microscope (green), scale bar = 200 μm. **c** Endometrium observed by hysteroscopy on postoperative day 35. **d**, **e** HE staining of porcine endometrial tissue sections, measuring endometrial thickness, *n* = 4 per group, ordinary one-way ANOVA with Tukey’s multiple comparison test. **f**, **g** Masson staining of porcine endometrial tissue sections, measuring collagen volume fraction, *n* = 4 per group, ordinary one-way ANOVA with Tukey’s multiple comparison test. **h**, **i** CK18 staining of porcine endometrial tissue sections, measuring glandular density, *n* = 4 per group, ordinary one-way ANOVA with Tukey’s multiple comparison test. **j**, **k** vWF staining of porcine endometrial tissue sections, measuring capillaries density, *n* = 4 per group, ordinary one-way ANOVA with Tukey’s multiple comparison test. **l**–**n** RT-PCR detection of *Col1*, *S100A8*, and *S100A9* mRNA levels in each group, *n* = 4 per group, ordinary one-way ANOVA with Tukey’s multiple comparison test. **o** Staining results for S100A8/A9 in porcine endometrial tissues, scale bar = 200 μm. Data are presented as means ± SD. *P < 0.05, **P < 0.01, ***P < 0.001 compared with indicated groups.

In this experiment, flow cytometry was used to identify the surface markers of MenSCs on P3. The results were consistent with the known characteristics of mesenchymal stem cells^12^, indicating that the cells used in the experiment were indeed MenSCs (Fig. 8a). They have a flattened shape and an elongated spindle-like appearance with a large nucleus and a small cytoplasm (Fig. 8b).

The hysteroscopic findings 35 days after electrocautery injury suggested that the endometrial damage caused by the electrocautery in the EI group led to the formation of scar tissue and dense bands of tissue in the uterine cavity. In contrast, the endometrial morphology in the EI+MenSCs group was similar to that of the normal control group, indicating that the injection of MenSCs may have prevented the formation of adhesions and promoted the regeneration of the endometrial tissue (Fig. 8c).

HE staining and Masson staining were used to evaluate the therapeutic effects of MenSCs on endometrial thickness and collagen deposition in pigs. The results showed that the endometrial thickness in the EI+MenSCs group (2.88 ± 0.48 mm) was similar to the control and sham groups (2.90 ± 0.58 mm, 2.76 ± 0.70 mm), while it was significantly thinner in the EI group (0.71 ± 0.14 mm) (Fig. 8d, e). In addition, the collagen fibers staining positive area was significantly increased in the EI group (62.0 ± 1.8%). In contrast, the positive area of collagen staining in the EI+MenSCs group (39.6 ± 4.4%) was similar to that in the control and sham groups (36.5 ± 1.9%, 34.5 ± 0.8%) (Fig. 8f, g).

The study utilized CK18 staining and vWF staining techniques to assess the impact of MenSCs treatment on endometrial glands and capillaries in a porcine model. The results demonstrated that the number of endometrial glands in the EI+MenSCs group (80.7 ± 6.1/view) was comparable to that of the control and sham groups (86.9 ± 23.0/view, 91.1 ± 5.8/view), while the number of endometrial glands in the EI group (34.0 ± 3.1/view) was significantly lower than the other three groups (Fig. 8h, i). Furthermore, the number of endometrial capillaries in the EI+MenSCs group (48.0 ± 10.2/view) was similar to that of the control and sham groups (45.5 ± 13.8/view, 45.0 ± 8.5/view), while the number of endometrial capillaries in the EI group (19.5 ± 4.7/view) was significantly lower than that of the other three groups (Fig. 8j, k).

To assess the impact of electrocautery injury and MenSCs treatment on the expression of Col1 and S100A8/A9 in porcine endometrium, we conducted RT-PCR and immunohistochemical analyses on the uterus in each group. The results showed that electrocautery injury significantly increased the relative expression of *Col1*, *S100A8*, and *S100A9* mRNA in porcine endometrium (Fig. 8l–n), while the mRNA levels of *Col1*, *S100A8*, and *S100A9* in the EI+MenSCs group were comparable to that of the control and sham groups (Fig. 8l–n). These results were further confirmed through immunohistochemistry (Fig. 8o).

To elucidate the associations among RAGE, p-JAK2, p-STAT3, and specific cell types within the in vivo lesion, we detected the expression of α-SMA, RAGE, p-JAK2, p-STAT3 in paraffin sections of endometrial tissues from the porcine IUA model by Immunohistochemistry. α-SMA, recognized as a biomarker of myofibroblasts, exhibited predominant expression within the endometrial stroma. Similarly, RAGE, p-JAK2, and p-STAT3 manifested expression within the endometrial stromal compartment, suggesting that the aforementioned targeted molecules may originate from myofibroblasts. Furthermore, an elevation in the expression levels of the aforementioned moleculars was distinctly observed within the EI group in contrast to both the CON and Sham groups. Conversely, a notable reduction in the expression of these molecules was evident following the administration of MenSCs treatment (Supplementary Fig. 2a).

In this study, the potential of MenSCs in preventing IUA was evaluated in a porcine model. The results showed that MenSCs treatment prevented the formation of scar tissue and dense bands of tissue in the uterine cavity, while also restoring endometrial thickness, and the number of glands and capillaries. Additionally, MenSCs treatment inhibited the expression of S100A8/A9, Col1, α-SMA, RAGE, p-JAK2, and p-STAT3 in porcine endometrium induced by electrocautery injury. These findings suggest that MenSCs treatment can effectively prevent endometrial injury-induced fibrosis and have the potential to prevent IUA formation by inhibiting the expression of S100A8/A9 in tissues.

## Discussion

The primary pathological feature of IUA is endometrial fibrosis^13^, which is characterized by the excessive activation and proliferation of myofibroblasts^14^. In normal tissue repair processes, local cytokines stimulate myofibroblast proliferation and activation, which then undergo apoptosis following tissue repair^15^. However, in pathological tissue fibrosis, myofibroblasts are overly activated, leading to the accumulation of ECM components, the destruction of normal tissue structure and function, and the rapid proliferation of myofibroblasts under the influence of numerous growth and inflammatory factors^16^. Consistent with previous research^17^, our study also observed a significant increase in the expression of Col1 and α-SMA in the endometrium of IUA patients compared to healthy individuals, suggesting the presence of endometrial fibrosis in IUA patients.

S100A8/A9 is commonly used as a biomarker of inflammation and is widely utilized in the diagnosis and prognosis evaluation of inflammatory diseases. Our results demonstrate a significant increase in the expression of S100A8/A9 in the endometrium of IUA patients. The upregulation of S100A8/A9 has also been observed in other fibrotic diseases. For example, S100A8/A9 is significantly upregulated in patients with idiopathic pulmonary fibrosis^5^ and is considered a reliable biomarker^18^. In acute kidney injury, S100A8/A9 initiates and amplifies the inflammatory response, while S100A8/A9 blockade improves renal function, reduces inflammatory monocyte infiltration, and prevents long-term renal fibrosis^19^. Furthermore, the expression of S100A8 and S100A9 is significantly increased on tubular epithelial cells in diabetic kidneys, and S100A8/A9 knockdown alleviates renal interstitial fibrosis in diabetic mice^20^. S100A8/A9 is primarily expressed in myeloid cells, including neutrophils^21^, monocytes^22,23^, activated macrophages^22^, and dendritic cells^24^, as well as in other secretory cells such as tissue epithelial cells^25^, endothelial cells^26^, and keratinocytes^27^. In normal physiological conditions, S100A8/A9 constitutes 45% and 5% of the soluble cytoplasmic protein content in neutrophils and monocytes, respectively^28,29^. Our immunofluorescence results revealed that the S100A8/A9-positive cells in the endometrial tissue of IUA patients were highly correlated with CD16-positive cells, indicating that S100A8/A9 was predominantly secreted by neutrophils.

Our in vitro experiments demonstrate that S100A8/A9 enhances the proliferation of hEnSCs in a concentration-dependent manner, and the effect of S100A8/A9 on hEnSCs proliferation is comparable to that of TGF-β, a well-established activator of stromal cells^30,31^. Given prior research indicating the ability of S100A8/A9 to provoke lung fibroblasts^5^ and keratinocytes^32^ into generating various inflammatory factors, we have posited that this protein complex may instigate similar outcomes in hEnSCs. Our findings reveal that S100A8/A9 is capable of triggering the upregulation of pro-inflammatory cytokines IL-1β and IL-6 in hEnSCs, thereby providing evidence for its pro-inflammatory effect.

The extracellular S100A8/A9 protein engages with various receptors, including RAGE and TLR4, located on the cell membrane surface, modulating cellular function through autocrine or paracrine mechanisms^4^. RAGE, a member of the immunoglobulin superfamily^33^, consists of extracellular, hydrophobic transmembrane, and cytoplasmic domains^34^, and participates in cell migration, invasion, survival, and apoptosis^35^. It is involved in the pathophysiology of various diseases, such as inflammatory and vascular diseases, and cancer^36–38^. As a multi-ligand receptor, RAGE binds to a range of ligands, including S100/Calgranulins, AGEs, and HMGB1/Amphoterin, eliciting oxidative stress and an inflammatory response^39–42^. TLR4, a key component of the mammalian innate immune system, triggers the immune system in response to pathogenic infection or tissue damage^43^. S100A8/A9 initiates an immune response to noninfectious inflammatory processes by binding to TLR4^44^. We therefore examined whether S100A8/A9-mediated activation of hEnSCs is dependent on RAGE and TLR4 receptors. Our findings reveal that S100A8/A9 increases the expression of RAGE protein in hEnSCs but does not affect TLR protein expression. Pretreatment of hEnSCs with a RAGE blocker significantly inhibits the effects of S100A8/A9 on promoting cell proliferation, inducing the expression of Col1 and α-SMA proteins, and activating the JAK2/STAT3 signaling pathway. However, treatment with a TLR4 blocker does not have a similar effect. Therefore, our data suggest that S100A8/A9 may promote fibrosis by binding to RAGE in hEnSCs.

RAGE is a pattern-recognition receptor that recognizes a variety of endogenous ligands and initiates several signaling cascades, including the JAK/STAT pathway, which has been implicated in the pathogenesis of inflammatory response^6^. Research on pulmonary fibrosis has demonstrated that JAK2/STAT3 signaling pathway activation is critical in inducing epithelial to mesenchymal and fibroblast to myofibroblast transitions^45^. Inhibitors of the JAK2/STAT3 pathway can effectively prevent fibroblast migration, reduce Bcl-2 expression, prevent fibroblast senescence, and alleviate impaired autophagy^46^. Various studies have shown that the activation of the JAK2/STAT3 signaling pathway is essential in fibrosis development in various organs such as the lung, kidney, and liver^45–47^. In our study, we have revealed that S100A8/A9 exerts a significant influence on the phosphorylation of the JAK2/STAT3 signaling pathway in hEnSCs. Notably, the promotion of S100A8/A9 on JAK2 and STAT3 phosphorylation was hindered upon pretreatment with RAGE inhibitor. Furthermore, the pro-fibrotic effects of S100A8/A9 were impeded after pretreatment with a JAK2/STAT3 pathway inhibitor. Collectively, these results propose that S100A8/A9 stimulates the activation of the JAK2/STAT3 signaling pathway through its interaction with RAGE. Subsequently, this activation triggers the proliferation of hEnSCs, leading to the upregulation of ECM expression and deposition, thereby accelerating the fibrosis process. It has been demonstrated that S100A9 induces the production of IL-6 through engagement with RAGE signaling^48^. As our prior investigations and sequencing outcomes similarly revealed an upregulation of IL-6 in IUA endometrium. Interestingly, IL-6 is reported to function as the primary upstream activator rather than the downstream target of the JAK2/STAT3 pathway^49,50^. Consequently, it is conceivable that IL-6, induced by activated RAGE through interaction with S100A8/A9, may activate the JAK2/STAT3 pathway via IL-6 receptors.

Due to ethical constraints, direct investigation of the pathological mechanism and experimental treatment of IUA in patients is difficult. Therefore, establishing an animal model of IUA is crucial for research purposes. Bama miniature pig, due to its anatomical and physiological similarities to humans, is an ideal model for this purpose^51^. Currently, electrocautery injury during operative hysteroscopy is one of the primary causes of IUA^52^. Thus, in this study, the electrocautery injury method was employed to establish an animal model of IUA in Bama miniature pig. The findings indicated that electrocauterization reduced endometrial thickness, decreased gland and microvessel numbers, and increased fibrosis proportion. After thirty-five days, hysteroscopy revealed classic IUA characteristics such as white scar tissue in the uterine wall and dense fiber cords in the uterine cavity, indicating that the electrocautery injury method successfully established an IUA model in the Bama miniature pig.

The use of MenSCs in the treatment of IUA shows promising results and has attracted increasing attention from researchers. The potential mechanisms underlying the therapeutic effects of MenSCs on IUA include the promotion of endometrial cell proliferation, acceleration of endometrial repair, and improvement of endometrial morphology and function^10,53^. Moreover, the current study suggests that MenSCs may exert their therapeutic effects by regulating S100A8/A9 and RAGE/JAK2/STAT3 signaling pathways. S100A8/A9 has been found to be upregulated in the endometrium of IUA pig and is closely related to the pathogenesis of IUA. The decrease in S100A8/A9 expression after MenSCs treatment suggests that MenSCs may modulate the expression of S100A8/A9 and contribute to the improvement of endometrial fibrosis and restoration of endometrial structure and function.

**Fig. 9:**
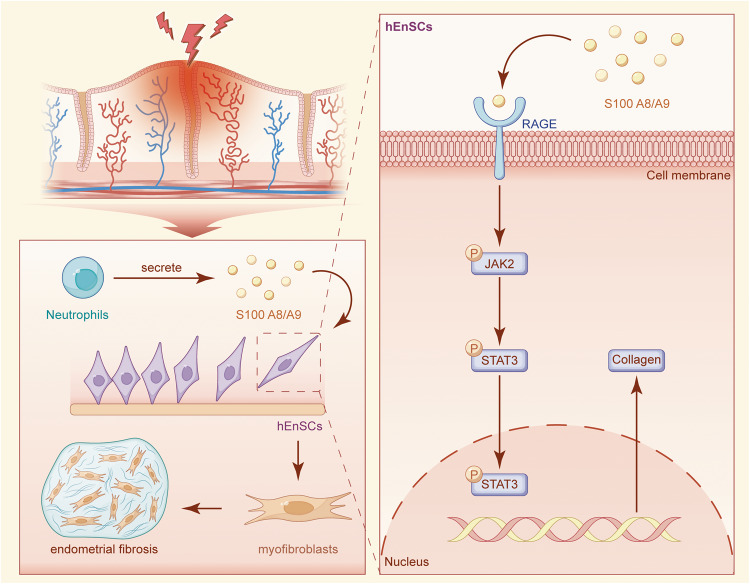
Schematic representation of the role of S100A8/A9 in activating hEnSCs. Following endometrial injury, neutrophils migrate to the trauma site, releasing S100A8/A9. S100A8/A9 binds to the extracellular domains of RAGE on the membrane of hEnSCs, activating the JAK2/STAT3 signaling pathway. Subsequently, it promotes the differentiation of hEnSCs into myofibroblasts, with increased expression of Col1 and α-SMA proteins.

In summary, the present investigation provides insights into the underlying mechanisms of endometrial fibrosis in patients with IUA, suggesting that S100A8/A9 is associated with endometrial fibrosis and IUA formation (Fig. 9). Our study indicates a significant increase in the expression levels of S100A8/A9 and the degree of endometrial fibrosis in IUA patients. Moreover, we demonstrate that S100A8/A9 promotes the differentiation of hEnSCs into myofibroblasts via the RAGE/JAK2/STAT3 signaling pathway, thus contributing to the formation of fibrosis. Furthermore, we establish a Bama miniature pig IUA model using the electrocautery injury method and suggest that MenSCs may reduce S100A8/A9, Col1, α-SMA, RAGE, p-JAK2, and p-STAT3 expression at the injury site, thereby alleviating endometrial fibrosis.

## Methods

### Patients and sample collection

This study was approved by the Ethics Committee of Shengjing Hospital of China Medical University (2019PS012F). All the volunteers signed an informed consent after fully understanding the experimental purpose and operating procedures. All ethical regulations relevant to human research participants were followed. Tissue samples were obtained from patients aged 20–45 years without uterine myoma, adenomyoma, endometriosis, or malignant tumor. The samples of IUA were from patients who underwent transcervical resection of adhesions for grade III or above IUA (European Society for Gynecologic Endoscopy classification of uterine adhesion). The samples (about 0.1 cm^3^) were collected from the scar tissue at the adhesion site by using a hysteroscopic cold knife. In addition, the normal endometrial tissues were collected from women without pathological abnormality during the hysteroscopy by using an endometrial curette.

### Culture and identification of hEnSCs

Primary normal hEnSCs were isolated from normal endometrium without endometriosis. The collected fresh tissues were washed 3–5 times with precooled PBS and then cut into 1-mm^3^ pieces. These pieces were digested with collagenase type I (Gibco, 0.1 mg/ml) for 1 h at 37 °C. Red blood cells were removed using red blood cell lysate reagent (Solarbio, China). The tissue homogenate was filtered through an aseptic 40-µm nylon strainer to remove undigested tissues and epithelial cells. The filtrate was centrifuged at 300 g for 5 min. After discarding the supernatant, hEnSCs were resuspended in DMEM containing 10% fetal bovine serum and then incubated in 5% CO_2_ at 37 °C. HEnSCs of passage 3 were collected for subsequent experiments.

### Cell proliferation assay

A Cell Counting Kit-8 (CCK-8) (DOJINDO, Japan) was used to assess cell viability in 96-well plates. After treatments, 10 μl of CCK-8 solution was added to each well and incubated for 2.5 h. Absorbance was determined at 450 nm using a microplate reader (BioTek, Winooski, VT, USA). The results are presented as a percent of the control results.

### PCR

Total RNA from the endometrium was isolated using TRIzol reagent according to the manufacturer’s protocol. Amplification was performed with a fast real-time PCR system (LightCycler480II, Roche, CHE) and a SYBR Premix Ex Taq Kit (TaKaRa, Dalian, China, #RR420L). PCR primer sequences (forward and reverse, respectively) were 5’-TATCATCGACGTCTACCACAAG-3’ and 5’-TCTGCACCCTTTTTCCTGATAT-3’ for human *S100A8*; 5’-CCTTCCACCAATACTCTGTGAA-3’ and 5’-GGTCCTCCATGATGTGTTCTAT-3’ for human *S100A9*; 5’-AAAGATGGACTCAACGGTCTC-3’ and 5’-CATCGTGAGCCTTCTCTTGAG-3’ for human *Col1*; 5’-CTTCGTTACTACTGCTGAGCGTGAG-3’ and 5’-CCATCAGGCAACTCGTAACTCTTCTC-3’ for human *α-SMA*; 5’-CTCTCCTCAAATCCACTGGATG-3’ and 5’-CTATCTCAGGGAGGATCAGCA-3’ for human *RAGE*; 5’-GACGAAGACTGGGTGAGGAATGAAC-3’ and 5’-CCTGGATGATGTTAGCAGCGATGG-3’ for human *TLR4*; 5’-GCCAGTGAAATGATGGCTTATT-3’ and 5’-AGGAGCACTTCATCTGTTTAGG-3’ for human *IL-1β*; 5’-CACTGGTCTTTTGGAGTTTGAG-3’ and 5’-GGACTTTTGTACTCATCTGCAC-3’ for human *IL-6*; 5’-AGCCCTGGTATGAGCCCATCTATC-3’ and 5’-TCCCAAAGTAGACCTGCCCAGAC-3’for human *TNFα*; 5’-CAGGAGGCATTGCTGATGAT-3’ and 5’-GAAGGCTGGGGCTCATTT-3’ for human *GAPDH*; 5’-TGCTGACGGATCTGGAGAGTGC-3’ and 5’-GCGTAGATGGCGTGGTAATTCCC-3’ for swine *S100A8*; 5’-GACCTGGACACTAATGTGGACAAGC-3’ and 5’-TCCTCGTGAGAAGCTACCGTCAG-3’ for swine *S100A9*; 5’-CTCAAGATGTGCCACTCCGACTG-3’ and 5’-GTCTCGCCTGTCTCCATGTTGC-3’ for swine *Col1*; 5’-GATTCCACCCACGGCAAGTTCC-3’ and 5’-GATTCCACCCACGGCAAGTTCC-3’ for swine *GAPDH*. mRNA levels were normalized to the expression of endogenous control GAPDH in triplicate and were calculated by the 2-ΔΔCt method.

### Western blot

A quantity of 10 μg of protein was loaded onto a 10% gradient polyacrylamide gel, electrophoretically transferred to a polyvinylidene difluoride membrane and probed with the following primary antibodies: S100A8/A9 (1:1000, Abcam, UK), Col1 (1:2000, Proteintech, China), α-SMA (1:6000, Proteintech, China), JAK2 (1:1000, Proteintech, China), p-JAK2 (Tyr1007) (1:1000, Absin, China), STAT3 (1:2000, Proteintech, China), p-STAT3(Tyr705) (1:1000, Absin, China), RAGE(1:1000, Proteintech, China), TLR4 (1:1000, Proteintech, China),  and GAPDH (1:1000, Absin, China), which was used as an internal control. Secondary antibodies were horseradish peroxidase-conjugated to mouse anti-rabbit/mouse IgG (1:5000, Proteintech, China).

### Immunohistochemistry and immunofluorescence

Immunohistochemistry was carried out by using S100A8/A9 (1:1000, Abcam, UK), Col1 antibody (1:1000, Proteintech, China), α-SMA (1:500, Proteintech, China), RAGE (1:200, Proteintech, China), p-JAK2(Tyr1007) (1:100, Absin, China), p-STAT3 (Tyr705) (1:100, Absin, China), CK18(1:1000, Proteintech, China), and vWF (1:1000, Proteintech, China). In brief, formalin-fixed, paraffin-embedded tissues were cut into 5-μm thickness and subjected to deparaffinization and rehydration. Following antigen retrieval, tissue sections were incubated with primary antibody overnight at 4 °C. After washed with PBS, sections were incubated with biotinylated secondary antibody for 1 h at room temperature. A DAB kit was employed as the chromogen and slides were counterstained with hematoxylin.

After deparaffinization, the sections were heated in EDTA for antigen retrieval and then blocked with BSA for 30 min at room temperature. The sections were then incubated with two kinds of primary antibodies at 4 °C overnight in a humidified chamber: CD16 antibody (1:5000, Servicebio, China) and S100A8/A9 antibody (1:5000, Abcam, UK). The next day, the sections were incubated with secondary antibodies for 50 min at room temperature. The secondary antibodies used were goat anti-rabbit Cy3 and goat anti-rabbit iF647 (both from Servicebio, China, diluted at 1:300). The nuclei were stained with DAPI for 10 min.

### Cell immunofluorescence

The cells were fixed with 4% paraformaldehyde, permeabilized in phosphate-buffered saline (PBS) containing 0.1% Triton X-100, and blocked in 10% goat serum albumin in PBS. The cells were incubated with one of the following antibodies: Col1 antibody (1:500, Proteintech, China), α-SMA antibody (1:200, Proteintech, China), STAT3 antibody (1:500 in PBS, Proteintech, China), GFP antibody (1:100, Proteintech, China), in combination with goat anti-rabbit secondary antibody conjugated with CoraLite594 (1:500, Proteintech, China). Nuclei were counterstained with DAPI (Solarbio, China).

### MenSCs isolation and culture

MenSCs isolation and culture were performed as we previously described^10^. Briefly, menstrual blood samples were collected on the second day of menses and transferred onto Ficoll, where they were fractionated using density-gradient centrifugation. The purified mononuclear cells obtained from the central cell layer were then washed and cultured in tissue culture bottles using Chang’s medium. The cells were incubated at 37 °C in an atmosphere containing 5% CO_2_, and the medium was changed every 3 days until the adherent cells reached 80–90% confluence. Finally, the cells were passaged using trypsin.

### Establishment of IUA model of Bama miniature pig and local injection of MenSCs into endometrium

Four 9-month-old female Bama miniature pigs from the Beijing ShiChuang miniature pig breeding base, weighing between 18 and 20 kg, were housed in a controlled environment with a temperature range of 20 °C ~24 °C, relative humidity of 40% ~ 70%, and a light cycle of 12 h light and 12 h dark. The pigs were given free access to food and water. Electrocautery injury was utilized to establish the Bama miniature pig IUA model. Each pig had one internal control with two completely separated horns; one horn served as the electrocautery injury group (EI, *n* = 4), and the other served as the normal control group (Control, *n* = 4). Prior to surgery, the pigs were fasted and deprived of water for 12 h. Tiletamine–Zolazepam injection (25 mg/Kg) was given by intramuscular injection for induction of anesthesia, and anesthesia was maintained with isoflurane through the respirator mask. A 5–8 cm longitudinal incision was made in the middle of the lower abdomen to expose the uterus, and one cornu uteri was selected as the EI group. The entire myometrium was longitudinally cut along the opposite side of the uterine mesangium, and a 1–2 cm incision was made to expose the endometrium. Electrocautery was used to damage the endometrium, and the intensity of electrocautery was set at 30 W, 40 W, 50 W, 60 W, and 70 W, with corresponding groups named EI30, EI40, EI50, EI60, and EI70, respectively. The electrotome was run at approximately 0.5 cm/s, and each part was electrocautered three times. The uterine incision was sutured with a 4-0 absorbable suture. The normal control group consisted of the untreated part of the contralateral uterine horn. The abdominal cavity was repeatedly rinsed with normal saline, and the abdomen was closed layer by layer. The target uterine tissue was excised by laparotomy at 7 days, 21 days, and 35 days after the electrocautery injury operation. One full-layer uterus with a thickness of approximately 0.5 cm was obtained from each group and fixed in paraformaldehyde. The endometrium was excised from the remaining tissue and stored in RNAlater storage solution at −80 °C for future analysis.

In the study investigating the potential of MenSCs in preventing IUA, a total of sixteen 9-month-old female Bama miniature pigs were included and categorized into four groups. The first group underwent electrocautery injury (EI) with a power of 70 W. The second group, EI+MenSCs, received electrocautery injury with 70 W power followed by multiple injections of 1 ml containing 2 × 10^6^ MenSCs into the endometrial layer at the site of injury. The third group was the sham group, in which the endometrium was exposed and sutured without any additional intervention. Lastly, the fourth group was the control group, which involved selecting the uterus without any treatment as the normal control group. Thirty-five days after electrocautery, endometrial images were captured using a flexible hysteroscope provided by L.H.^54^.

All experimental procedures were approved by the Animal Experimental Ethics Committee of Shengjing Hospital of China Medical University(2021PS529k). We have complied with all relevant ethical regulations for animal use.

### Endometrial thickness measurement, endometrial gland, and capillary count

H&E-stained slices were photographed, and the endometrial thickness was then measured from four directions of each uterine cross-section using Image J software to calculate the mean thickness. CK18-stained sections were photographed, and the number of glands was counted in six randomly selected fields. vWF-stained sections were also photographed, and the number of capillaries was counted in six randomly selected fields. Complete brown endothelial clusters and brown epithelial cell clusters (positive for vWF and CK18 immunohistochemistry, respectively) were counted as independent capillaries and glands, respectively.

### Masson staining and determination of collagen volume fraction (CVF) in endometrium

Masson’s trichrome staining was performed according to the kit manufacturer’s instructions (Beijing Solarbio Science and Technology Co., Ltd., Beijing, China). Six fields were randomly selected for each Masson section. Image J software was used to calculate the percentage of the dark blue area of collagen staining positive in each field relative to the total tissue area, and the average value was taken.

### Statistics and reproducibility

All data were analyzed with GraphPad Prism version 8.0 (GraphPad Prism Software, Inc., CA, USA) and are presented as means ± SD. Comparisons between two groups were performed using an unpaired two-tailed *t* test or unpaired *t* test with Welch’s correction. Comparisons between three or more groups were performed using ordinary one-way ANOVA with Tukey’s or Dunnett’s multiple comparison test, Welch’s ANOVA test with Dunnett’s T3 or Tamhane’s T2 multiple comparisons test. A *P* value < 0.05 was considered to be statistically significant. To ensure reproducibility, a minimum of three replicates were conducted. The figure legend details sample sizes and specifies the replicates.

### Reporting summary

Further information on research design is available in the Nature Portfolio Reporting Summary linked to this article.

### Supplementary information

Supplementary Information

Description of Supplementary Materials

Supplementary Data 1

Reporting-Summary

## Data Availability

The uncropped/unedited western blot images are included in Supplementary Fig. 3. The source data can be obtained from Supplementary Data 1, while other data can be obtained from the corresponding author upon reasonable request.
