# Supplementary material for: S100A8/A9 promotes endometrial fibrosis via regulating RAGE/JAK2/STAT3 signaling pathway
Source: Commun Biol. 2024 Jan 22;7:116. doi: 10.1038/s42003-024-05814-5 (PMC10803310; doi:10.1038/s42003-024-05814-5)
Supplement: Supplementary file 1 — Supplementary Information [file 42003_2024_5814_MOESM1_ESM.pdf]

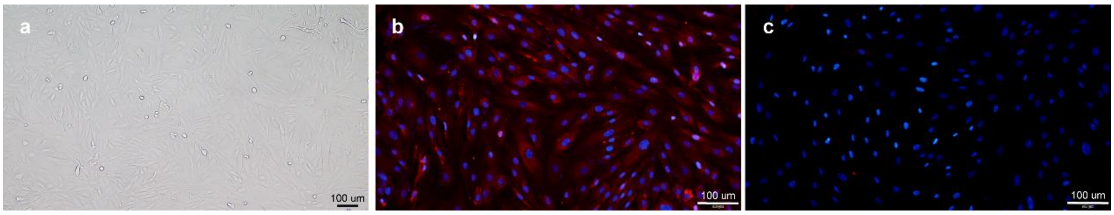

Supplementary Fig. 1 Representative optical microscope images and immunofluorescent staining of P3 hEnSCs. a Representative optical microscope images of P3 hEnSCs. **b** Immunofluorescence image of Vimentin staining (blue: DAPI, red: Vimentin). **c** Immunofluorescence image of α-SMA staining (blue: DAPI, red: α-SMA). Scale bar = 100 μm.

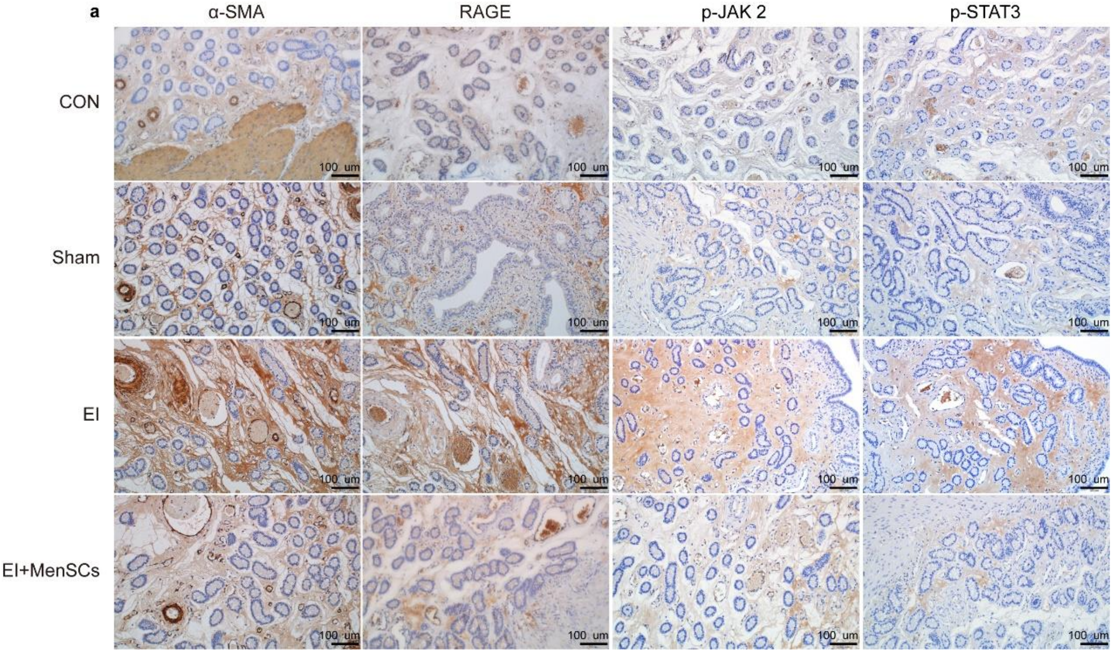

Supplementary Fig. 2 Representative immunohistochemistry images of α-SMA, RAGE, p-JAK2, and p-STAT3 in porcine endometrial tissue sections from CON, Sham, EI and EI+MenSCs groups are shown. Scale bar = 100 μm.

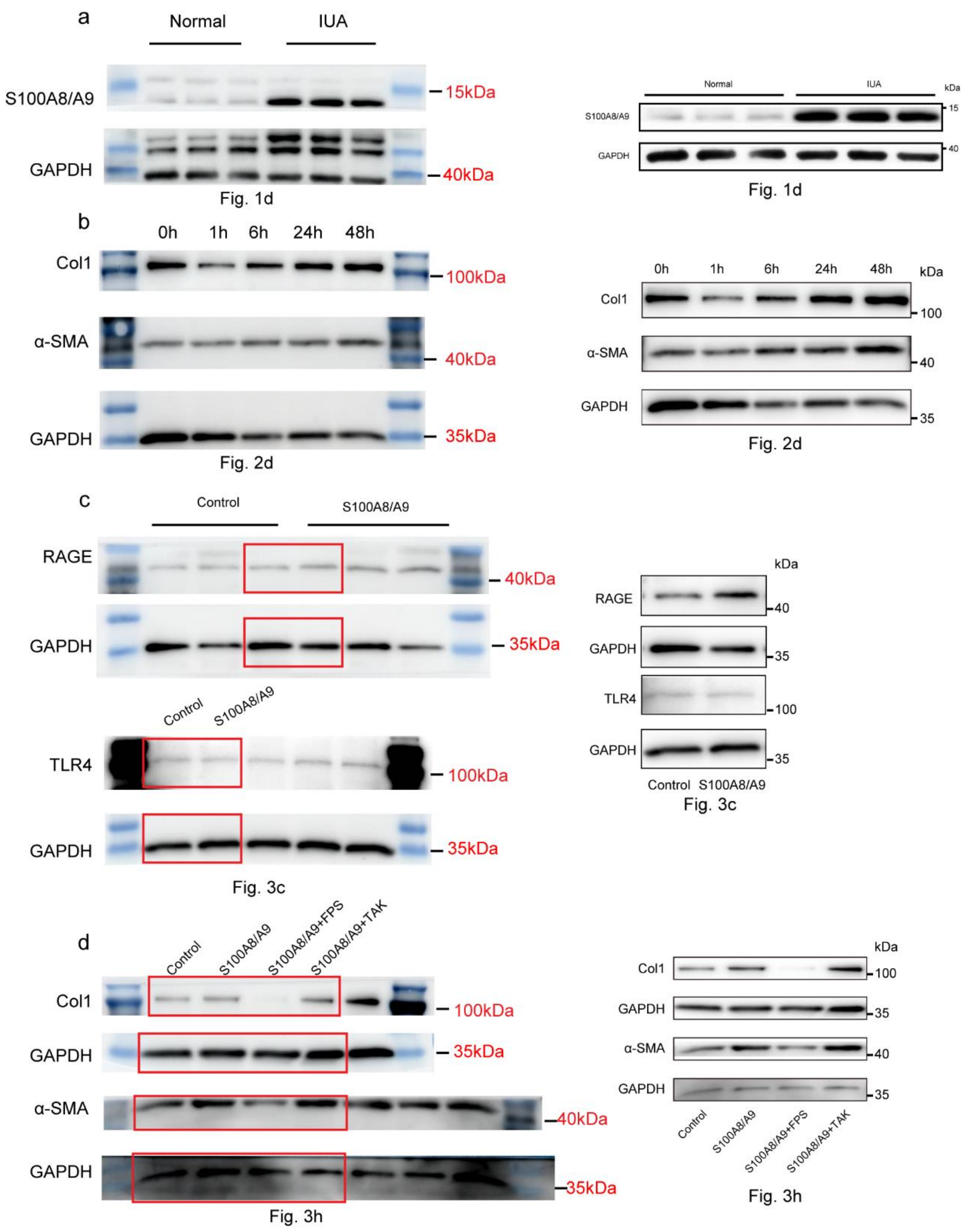

Supplementary Fig. 3 Uncropped scans of Western blot results.

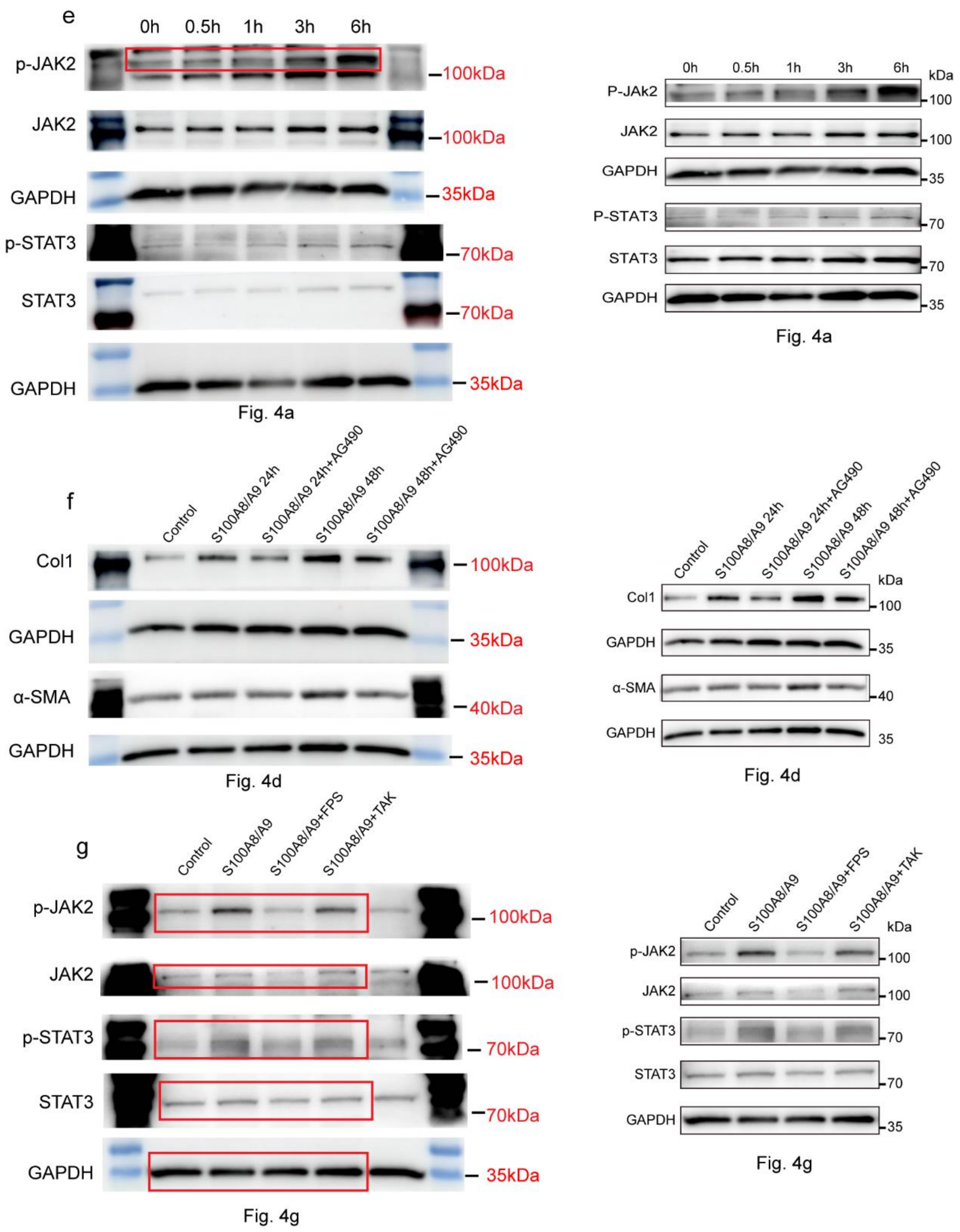

Supplementary Fig. 3 Uncropped scans of Western blot results.
