# Supplementary material for: S100A8/A9 promotes endometrial fibrosis via regulating RAGE/JAK2/STAT3 signaling pathway
Source: Commun Biol. 2024 Jan 22;7:116. doi: 10.1038/s42003-024-05814-5 (PMC10803310; doi:10.1038/s42003-024-05814-5)
Supplement: Supplementary file 4 — Reporting-Summary [file 42003_2024_5814_MOESM4_ESM.pdf]

nature portfolio

Corresponding author(s): Jichun Tan

Last updated by author(s): Dec 26, 2023

Reporting Summary

Nature Portfolio wishes to improve the reproducibility of the work that we publish. This form provides structure for consistency and transparency in reporting. For further information on Nature Portfolio policies, see our Editorial Policies and the Editorial Policy Checklist.

Statistics

For all statistical analyses, confirm that the following items are present in the figure legend, table legend, main text, or Methods section.

n/a	Confirmed	
☐	☒	The exact sample size (*n*) for each experimental group/condition, given as a discrete number and unit of measurement
☐	☒	A statement on whether measurements were taken from distinct samples or whether the same sample was measured repeatedly
☐	☒	The statistical test(s) used AND whether they are one- or two-sided<br>*Only common tests should be described solely by name; describe more complex techniques in the Methods section.*
☒	☐	A description of all covariates tested
☐	☒	A description of any assumptions or corrections, such as tests of normality and adjustment for multiple comparisons
☐	☒	A full description of the statistical parameters including central tendency (e.g. means) or other basic estimates (e.g. regression coefficient) AND variation (e.g. standard deviation) or associated estimates of uncertainty (e.g. confidence intervals)
☐	☒	For null hypothesis testing, the test statistic (e.g. *F*, *t*, *r*) with confidence intervals, effect sizes, degrees of freedom and *P* value noted<br>*Give P values as exact values whenever suitable.*
☒	☐	For Bayesian analysis, information on the choice of priors and Markov chain Monte Carlo settings
☒	☐	For hierarchical and complex designs, identification of the appropriate level for tests and full reporting of outcomes
☒	☐	Estimates of effect sizes (e.g. Cohen's *d*, Pearson's *r*), indicating how they were calculated

Our web collection on statistics for biologists contains articles on many of the points above.

Software and code

Policy information about availability of computer code

Data collection	GraphPad Prism version 8.0
Data analysis	GraphPad Prism version 8.0

For manuscripts utilizing custom algorithms or software that are central to the research but not yet described in published literature, software must be made available to editors and reviewers. We strongly encourage code deposition in a community repository (e.g. GitHub). See the Nature Portfolio guidelines for submitting code & software for further information.

Data

Policy information about availability of data

All manuscripts must include a data availability statement. This statement should provide the following information, where applicable:

- Accession codes, unique identifiers, or web links for publicly available datasets
- A description of any restrictions on data availability
- For clinical datasets or third party data, please ensure that the statement adheres to our policy

The data that support the findings of this study are available on request from the corresponding author, [Jichun Tan], upon reasonable request.

Research involving human participants, their data, or biological material

Policy information about studies with human participants or human data. See also policy information about sex, gender (identity/presentation), and sexual orientation and race, ethnicity and racism.

Reporting on sex and gender	The study exclusively involved female participants, and no analyses were conducted with regards to sex or gender
Reporting on race, ethnicity, or other socially relevant groupings	The manuscript does not make any explicit references to race, ethnicity, or other socially relevant groupings.
Population characteristics	See above
Recruitment	The recruitment of all volunteers was exclusively limited to outpatients.
Ethics oversight	This study was approved by the Ethics Committee of Shengjing Hospital of China Medical University (2019PS012F).

Note that full information on the approval of the study protocol must also be provided in the manuscript.

Field-specific reporting

Please select the one below that is the best fit for your research. If you are not sure, read the appropriate sections before making your selection.

☒ Life sciences ☐ Behavioural & social sciences ☐ Ecological, evolutionary & environmental sciences

For a reference copy of the document with all sections, see nature.com/documents/nr-reporting-summary-flat.pdf

Life sciences study design

All studies must disclose on these points even when the disclosure is negative.

Sample size	No statistical methods were used to pre-determine sample sizes, and the sample size was similar to those reported in previous publications with similar methodologies.
Data exclusions	No data were excluded from the analyses
Replication	All studies were conducted according to the recently published NIH guidelines on reproducibility and rigor in scientific research.
Randomization	The experimental subjects were allocated into groups using the random number table method
Blinding	Investigators were blinded to group allocation during data collection and anaysis.

Reporting for specific materials, systems and methods

We require information from authors about some types of materials, experimental systems and methods used in many studies. Here, indicate whether each material, system or method listed is relevant to your study. If you are not sure if a list item applies to your research, read the appropriate section before selecting a response.

Materials & experimental systems

n/a	Involved in the study	
☐	☒	Antibodies
☒	☐	Eukaryotic cell lines
☒	☐	Palaeontology and archaeology
☐	☒	Animals and other organisms
☒	☐	Clinical data
☒	☐	Dual use research of concern
☒	☐	Plants

Methods

n/a	Involved in the study	
☒	☐	ChIP-seq
☐	☒	Flow cytometry
☒	☐	MRI-based neuroimaging

Antibodies

Antibodies used	Anti-S100A8/A9 antibody (abcam, ab22506, GR3338633-7)<br>Anti-Col1 (proteintech, 14695-1-AP, 00105556)<br>Anti-α-SMA antibody (proteintech, 14395-1-AP, 00118974)

	Anti-JAK2 antibody (proteintech, 17670-1-AP, 00046735)<br>Anti-STAT3 antibody (proteintech, 10253-2-AP, 00097602)<br>Anti-p-JAK2 (Tyre1007) antibody (absin, abs130650, O15P)<br>Anti-p-STAT3(Tyr705) antibody (absin, abs130918, AX27)<br>Anti-RAGE antibody (proteintech, 66833-1-Ig, 10007561)<br>Anti-TLR4 antibody (proteintech, 66350-1- Ig, 10005089)<br>Anti- CK18 antibody （proteintech, 10830-1-AP, 00050736)<br>Anti-vWF antibody （proteintech, 11778-1-AP, 00095043）<br>Anti-GAPDH antibody (Absin, abs830030-100, M05A04)<br>Anti-CD16 antibody (Servicebio, GB113152-100, CR2309050)
Validation	We have specifically validated antibodies

Animals and other research organisms

Policy information about studies involving animals; ARRIVE guidelines recommended for reporting animal research, and Sex and Gender in Research

Laboratory animals	9-month-old female Bama miniature pigs
Wild animals	This study did not involve wild animals
Reporting on sex	Only female Bama miniature pigs were selected
Field-collected samples	The animals were fed in a controlled environment with temperature of 20℃~24℃, relative humidity of 40%~70% and light (12 h light and 12 h dark), with free access to food and water.
Ethics oversight	All experimental procedures were approved by the Animal Experimental Ethics Committee of Shengjing Hospital of China Medical University(2021PS529k).

Note that full information on the approval of the study protocol must also be provided in the manuscript.

Flow Cytometry

Plots

Confirm that:

☒ The axis labels state the marker and fluorochrome used (e.g. CD4-FITC).

☐ The axis scales are clearly visible. Include numbers along axes only for bottom left plot of group (a 'group' is an analysis of identical markers).

☐ All plots are contour plots with outliers or pseudocolor plots.

☒ A numerical value for number of cells or percentage (with statistics) is provided.

Methodology

Sample preparation	P3 generation MenSCs were digested with 0.25% trypsin, cell precipitates were collected, washed once with PBS and resuspended in PBS containing 0.5% BSA, and the antibodies were incubated according to the instructions, followed by assays
Instrument	FACScaliburTM Flow Cytometer (Becton-Dickinson, Mountain View, CA, USA)
Software	CellQuest software (Becton-Dickinson)
Cell population abundance	After selecting the cell population, 10,000 cells were counted for each indicator and the positivity rate was calculated
Gating strategy	Adjust the compensation after selecting the live cell population, set the fluorescence intensity at 4 powers of 10 to be positive, and count the proportion of positive cells for each indicator

☐ Tick this box to confirm that a figure exemplifying the gating strategy is provided in the Supplementary Information.
